# I_MDS: an inflammatory bowel disease molecular activity score to classify patients with differing disease-driving pathways and therapeutic response to anti-TNF treatment

**DOI:** 10.1371/journal.pcbi.1006951

**Published:** 2019-04-30

**Authors:** Stelios Pavlidis, Calixte Monast, Matthew J. Loza, Patrick Branigan, Kiang F. Chung, Ian M. Adcock, Yike Guo, Anthony Rowe, Frédéric Baribaud

**Affiliations:** 1 Janssen Research & Development Ltd, High Wycombe, United Kingdom; 2 National Heart and Lung Institute, Imperial College & Biomedical Research Unit, Royal Brompton & Harefield NHS Trust, London, United Kingdom; 3 Data Science Institute, Imperial College London, London, United Kingdom; 4 Janssen Research & Development LLC, United States of America; Norwegian University of Science and Technology, NORWAY

## Abstract

Crohn’s disease and ulcerative colitis are driven by both common and distinct underlying mechanisms of pathobiology. Both diseases, exhibit heterogeneity underscored by the variable clinical responses to therapeutic interventions.

We aimed to identify disease-driving pathways and classify individuals into subpopulations that differ in their pathobiology and response to treatment.

We applied hierarchical clustering of enrichment scores derived from gene set variation analysis of signatures representative of various immunological processes and activated cell types, to a colonic biopsy dataset that included healthy volunteers, Crohn’s disease and ulcerative colitis patients. Patient stratification at baseline or after anti-TNF treatment in clinical responders and non-responders was queried. Signatures with significantly different enrichment scores were identified using a general linear model. Comparisons to healthy controls were made at baseline in all participants and then separately in responders and non-responders. Fifty-nine percent of the signatures were commonly enriched in both conditions at baseline, supporting the notion of a disease continuum within ulcerative colitis and Crohn’s disease. Signatures included T cells, macrophages, neutrophil activation and poly:IC signatures, representing acute inflammation and a complex mix of potential disease-driving biology. Collectively, identification of significantly enriched signatures allowed establishment of an inflammatory bowel disease molecular activity score which uses biopsy transcriptomics as a surrogate marker to accurately track disease severity. This score separated diseased from healthy samples, enabled discrimination of clinical responders and non-responders at baseline with 100% specificity and 78.8% sensitivity, and was validated in an independent data set that showed comparable classification. Comparing responders and non-responders separately at baseline to controls, 43% and 70% of signatures were enriched, respectively, suggesting greater molecular dysregulation in TNF non-responders at baseline. This methodological approach could facilitate better targeted design of clinical studies to test therapeutics, concentrating on patient subsets sharing similar underlying pathobiology, therefore increasing the likelihood of clinical response.

## Author summary

Patients exhibiting similar phenotypical characteristics, diagnosed with the same disease, exhibit variable response to therapeutics. This is a major health care issue, due to the increased patient suffering and the socioeconomical burden that occurs. Crohn’s disease and ulcerative colitis constitute good examples of inflammatory conditions, with sufferers responding differentially to existent therapeutics. Here, we identified disease-driving pathways and classified individuals into subpopulations that differ in their pathobiology and response to treatment. We utilized gene set variation analysis and transcriptomic data from inflammatory bowel disease sufferers to stratify patients at baseline or after anti-TNF treatment in clinical responders and non-responders. We explored gene signatures obtained from the literature, relevant to immune processes, which were significantly enriched in disease compared to healthy controls, as well as before and after treatment. Using these signatures, we established an inflammatory bowel disease molecular activity score, which allowed us to separate clinical responders and non-responders at baseline with high specificity and sensitivity. We validated the proposed approach in an independent data set, demonstrating comparable classification. This methodological approach may lead to better targeted design of clinical studies, allowing the selection of patient sharing similar underlying pathobiology, thus increasing the likelihood of clinical response to treatment.

## Introduction

Inflammatory bowel disease (IBD) is a phenotypically and molecularly heterogeneous condition characterized by chronic inflammation of the gut [1, 2, 3, 4]. IBD patients unable to find effective therapy, experience an extremely poor quality of life and can progress to surgical removal of affected tissues [1, 3]. While controlling inflammation with relevant therapeutics has been shown to improve quality of life and clinical outcomes [5, 6], biomarkers to guide the choice of therapeutics are currently limited to CRP and fecal calprotectin, and patients must often be treated for an extended period to determine if the chosen drug is efficacious [7]. Considering the size of phase 2 and phase 3 studies in IBD and the increasingly routine inclusion of biomarker collections it might seem surprising that biomarkers for clinical response have not been identified. However, availability of data is not the main issue hampering personalized medicine in IBD. Personalized medicine, especially in Crohn’s disease (CD), is challenged by the lack of accuracy in defining a responsive phenotype and lack of agreement in the field on the types of molecular features that should be used to predict patient response [8, 9].

One of the most critical issues hampering identification of biomarkers for clinical response is the definition of a responder phenotype. Traditionally, CD and UC disease activity scores are based on patient reported outcomes, physician assessments, and, endoscopic assessment [1, 3]. While these endpoints are sufficiently accurate to assess efficacy of candidate therapeutics in clinical trials, they require relatively large numbers of subjects in each arm to account for variability. While there is likely bona fide variation in disease severity, the underlying molecular dysregulation remains unknown given that spontaneous and permanent remission is extremely rare. This variability in disease severity that does not correlate with the underlying molecular disease, challenges identification of biomarkers to guide treatment decisions because identification of these biomarkers is predicated on a well-defined and accurate response phenotype. In other words, all responders, post-treatment should be those that improved due to the specific mechanism of action of the drug. Introduction of even a small number of subjects who did not truly respond to the drug into the responder group will force computational methods to attempt to find biomarkers that apply to both bona fide and erroneous responders, alike. This will result in a reduced ability to identify the best biomarkers. In addition, responders and non-responders incorrectly identified clinically will falsely reduce sensitivity and specificity of a good biomarker because even a perfect biomarker will not agree with an erroneous clinical responder classification. Thus, the issues of defining response and identifying biomarkers for personalized medicine are inexorably linked.

Even with a perfect response phenotype there remains the challenge of identifying the correct tissue and the correct type of molecular data for biomarker identification. The main candidates have historically been gut tissue, the periphery, and genetics [10, 11, 12, 13, 14]. Gut tissue is attractive because this is the site of disease, however, many drugs are administered systemically so it is unclear to what degree gut tissue contains information regarding the capability of the individual to respond. The opposite argument can be made for the periphery. Indeed, it is easier to sample and captures the systemic state of the individual, while it may however not efficiently store information regarding the local state of the gut. Lastly, genetics, especially genome-wide approaches, presents the challenge of having enough individuals to identify associations [15]. In general, genetic associations with phenotypes in IBD have been weak compared to other diseases, though there are some hints that this approach may be fruitful [16, 17].

To address these challenges to personalized medicine in IBD we made several assumptions in the present work: 1) the response phenotype must be determined as accurately as possible; 2) the site of disease must contain information regarding the response potential of the individual and 3) both pre- and post-treatment samples are necessary to establish the molecular impact of the drug and in doing so evaluate the accuracy of the response phenotype. Thus, we used an established IBD dataset with rigorous response phenotype definitions to test an approach to capturing the molecular severity of disease [18]. Our results suggest that disease severity can be accurately tracked using biopsy transcriptomics in a manner that may support reducing the size of clinical trials designed to test the efficacy of therapeutics. Furthermore, our results suggest that response to anti-TNF may be related to the degree to which subjects are molecularly inflamed when the drug is administered. While it is widely assumed that activity of the TNF pathway would indicate those more likely to respond to anti-TNF therapy (we assume thanks to the evolution of personalized medicine in oncology) [19, 20], our results suggest the opposite conclusion. Specifically, that subjects with elevated activity of a particular pathway are less likely to respond to inhibition of that pathway. We hypothesize that this is due to pharmacological limitations in those patients such that drug levels cannot be increased to the point where signaling through the pathway is reduced sufficiently for clinical response.

## Materials and methods

### Gene signature collection

A collection of gene signatures was assembled from publications presenting results of microarray experiments relevant to immune system processes and responses. In general, these signatures include immune cell gene expression profiles from healthy and disease groups and gene expression changes in response to inflammatory modulators, such as cytokines and drugs. S1 Table provides an overview of the assembled gene signature collection while S2 Table reports the genes included in each signature. Gene signatures published in the original manuscripts were incorporated into our collection as presented by the authors. For other signatures in our collection, which were not presented in the original manuscript, differential gene expression analysis was performed using general linear modeling (GLM) of the published microarray datasets. Gene signatures were split into up- and down-regulated genes relative to control and sets of less than 5 genes were discarded. HGNChelper R package was used to automatically update outdated gene symbols [21]. The naming of signatures was based on the following approach: the tissue or cell type, followed by the experimental condition, organism, experimental setting (i.e. in vivo (IVV), in vitro (IVS) or ex vivo (EXV) stimulation), followed by the direction of expression (up or down). The signatures are listed in order of their acquisition (S1 and S2 Tables).

When comparing all gene lists from the 103 gene signatures to identify possible gene overlaps, we found that only 0.5% (28 of the 5253) of pairwise signature comparisons showed an overlap of 20% or more genes. Thus, these gene signatures predominantly represent lists of genes that are distinct from each other.

### Gene set variation analysis (GSVA)

GSVA was run using the R Bioconductor GSVA package [22]. Enrichment scores (ES) for gene expression data corresponding to Crohn’s disease (CD) and Ulcerative colitis (UC) ((GEO), GSE16879, [18]) were subjected to statistical analysis with the Array Studio software (Omicsoft Corp., Research Triangle Park, NC, USA). A GLM was applied to compare enrichment scores between groups. Reported values of significance (p) were adjusted with the Benjamini–Hochberg False Discovery Rate (FDR-BH) procedure [23].

Gene signatures ES were significantly different between two groups if the difference in means of the ES was at least 0.2 and the significance as measured by a t-test was less than or equal to 0.05.

Visualization of the distribution of ES was performed with the ggplot2 R package [24]. ES were subjected to hierarchical clustering and visualization utilizing the Euclidian distance metric and the Bioconductor ggplots R package [25].

### Analysis workflow

**Fig 1:**
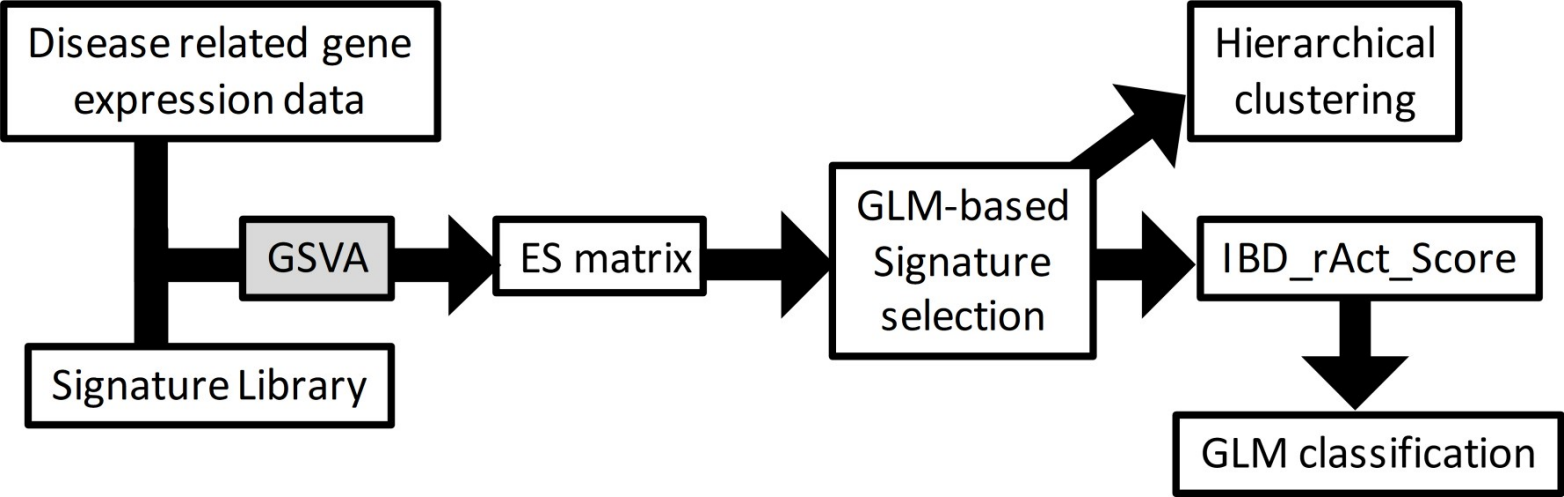
Workflow chart used to identify patient level disease driving biology. Disease related gene expression data representing the differential between either diseased to healthy or pretreated to post-treated samples for either CD or UC were assembled. Also assembled was a gene expression signature library representing various pathways or cell types. Gene set variation analysis (GSVA) was then applied to generate an enrichment score (ES) matrix in each disease which then underwent general linear model-based clustering to enable interpretation.

Fig 1 describes the workflow used to analyse the disease data sets using the assembled signature collection. An ES matrix was generated using GVSA applied to the data sets using the generated signature collection. GLM analysis was used to select significantly enriched signatures at baseline when comparing disease groups to NHV common to both diseases. The ES of these signatures were hierarchically clustered, and the ES for each of these signatures summed to build a molecular disease severity score. The molecular disease severity score was used to construct classification models using a GLM. For differential gene expression analysis, we defined a fold change of 2 and a falls discovery rate of 5% (FDR<0.05) as thresholds, using the entire gene expression platform. Following these criteria, 103 signatures were generated totaling 4322 unique genes. For the I_MSD score, 58 signatures consisting of 3055 unique genes were used.

### Ethics statement

For GSE16879: The study was carried out at the University Hospital of Gasthuisberg in Leuven (ClinicalTrials.gov number, NCT00639821). The ethics committee of the University Hospital approved the study and all individuals gave written informed consent. For GSE23597: The multicenter, randomized, double-blind, placebo-controlled ACT-1 (Clinical Trials.gov number, NCT00036439) was conducted globally at 62 (ACT-1) sites between March 2002 and March 2005. The Institutional Review Board or Ethics Committee at each site approved the protocols.

## Results

### Generation of the gene signature library

Previous analyses of transcriptomic data of biopsies taken from subjects with IBD has generally relied on the analysis of individual genes [18, 26, 27, 28]. Here, we sought to take a different approach and analyze previously published IBD data using gene expression signatures. Use of these signatures reduces the complexity of a gene expression dataset from over 50 thousand probe sets to 103 defined units of biology represented by each signature. The gene signature library we assembled from GEO datasets representing various pathways or cell types (S1 and S2 Tables) covering a broad range of immunological processes that could quantify IBD disease biology. These gene signatures can be used to explore patient stratification and biomarkers of disease severity with the GSVA algorithm which computes enrichment scores for each sample and each gene signature. This enrichment score is an estimate of the relative degree to which a sample expresses the genes in a given signature.

### Disease related gene expression and signature library data

Transcriptional data from IBD tissue was available through the gene expression omnibus (GEO) dataset GSE16879 from Crohn’s disease (CD) and ulcerative colitis (UC) [18]. This data set was chosen because it represented enough numbers of patients to assess patient stratification and it included patient data before and after a therapeutic intervention (anti-TNF therapy), yielding clinical responders (R) and non-responders (NR). In addition, the patients are clinically characterized in detail and criteria for identifying responders and non-responders well documented [18].

### Enriched signatures in Crohn’s disease and ulcerative colitis at baseline

**Fig 2:**
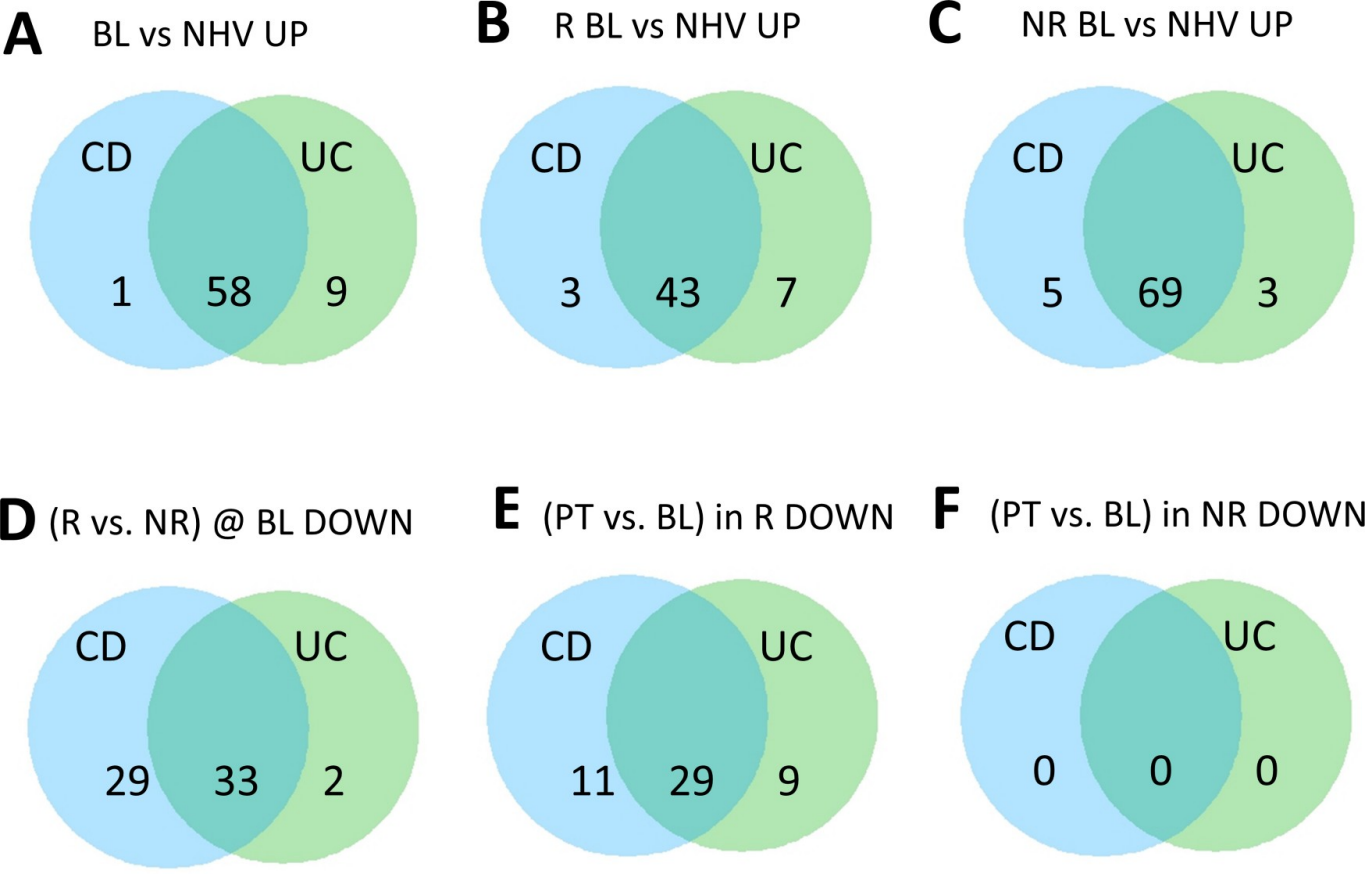
Differential signatures in Crohn’s disease and ulcerative colitis across various patient group comparisons. Venn diagrams of upregulated signatures significantly enriched using a general linear model analysis on GSVA ES comparing at baseline (BL) either all (A) or clinical responder (R) (B) or clinical non-responder (NR) (C) participant samples in CD and UC to healthy volunteers. Also shown are the results comparing R vs NR at BL (D), post-treatment (PT) vs BL in R (E) and NR (F) respectively. In A, B and C, and in D, E and F the number of signatures positively and negatively enriched are listed respectively.

We first assessed which gene signatures showed higher enrichment in each disease by comparing baseline enrichment scores for UC and CD samples to healthy controls (Figs 2 and S1). Signatures identified in this manner represent biological processes that may be dysregulated in disease. This analysis identified 59 signatures in CD and 67 signatures in UC that differed from healthy. 58 of those signatures overlapped between the two lists, suggesting that despite the clinical differences between the two diseases, biological processes in diseased tissues for CD and UC are highly concordant. This signature-based approach allows for the possibility that while different genes may be involved in specific immunological processes between the two diseases, the immunological processes themselves may be conserved. Notably, all 43 signatures that showed higher enrichment in CD responders, at baseline, compared to the healthy volunteers, were contained within the aforementioned 58 signatures.

The biology implicated by the 58 signatures shared between CD and UC was diverse. These signatures were derived from activated T cells, monocytes, macrophages and neutrophils but also from mouse lungs upon either poly IC or bleomycin treatment (S3 Table). Generally, many signatures represented acute inflammatory processes. Of note, several signatures derived from asthma samples from the lungs were also enriched (S3 Table) highlighting conserved disease mechanisms across various inflammatory diseases.

Next, we used our gene signature approach to explore whether our gene signatures changed with anti-TNF treatment. We first stratified our population based on whether or not a clinical response was achieved post-treatment as this factor may influence the observed molecular changes. Interestingly, in the R population, a total of 40 gene signatures for CD and 38 gene signatures for UC were found to be significantly down-regulated with anti-TNF treatment between baseline and post-treatment. All 38 UC gene signatures were part of the 40 CD downregulated signatures, suggesting that clinical benefit is associated with resolution of the same types of gene signatures for both diseases. This is consistent with our earlier observation that the same gene signatures also seem to be present at baseline in both diseases (Fig 1A). Indeed, the signatures that changed in the R population were a subset of those that were differentially enriched at baseline compared to healthy (S3 Table). Finally, the NR population had no significant molecular changes with our approach. Altogether these results suggested that clinical benefit may be coupled to molecular changes related to the same molecular dysregulation seen at baseline. Furthermore, this molecular dysregulation is highly conserved between CD and UC (S3 Table). Our approach appears to preferentially highlight molecular changes associated with the clinical state of the disease considering that pharmacodynamic changes associated with anti-TNF treatment were not detected in the NR population.

### Hierarchical cluster analysis

**Fig 3:**
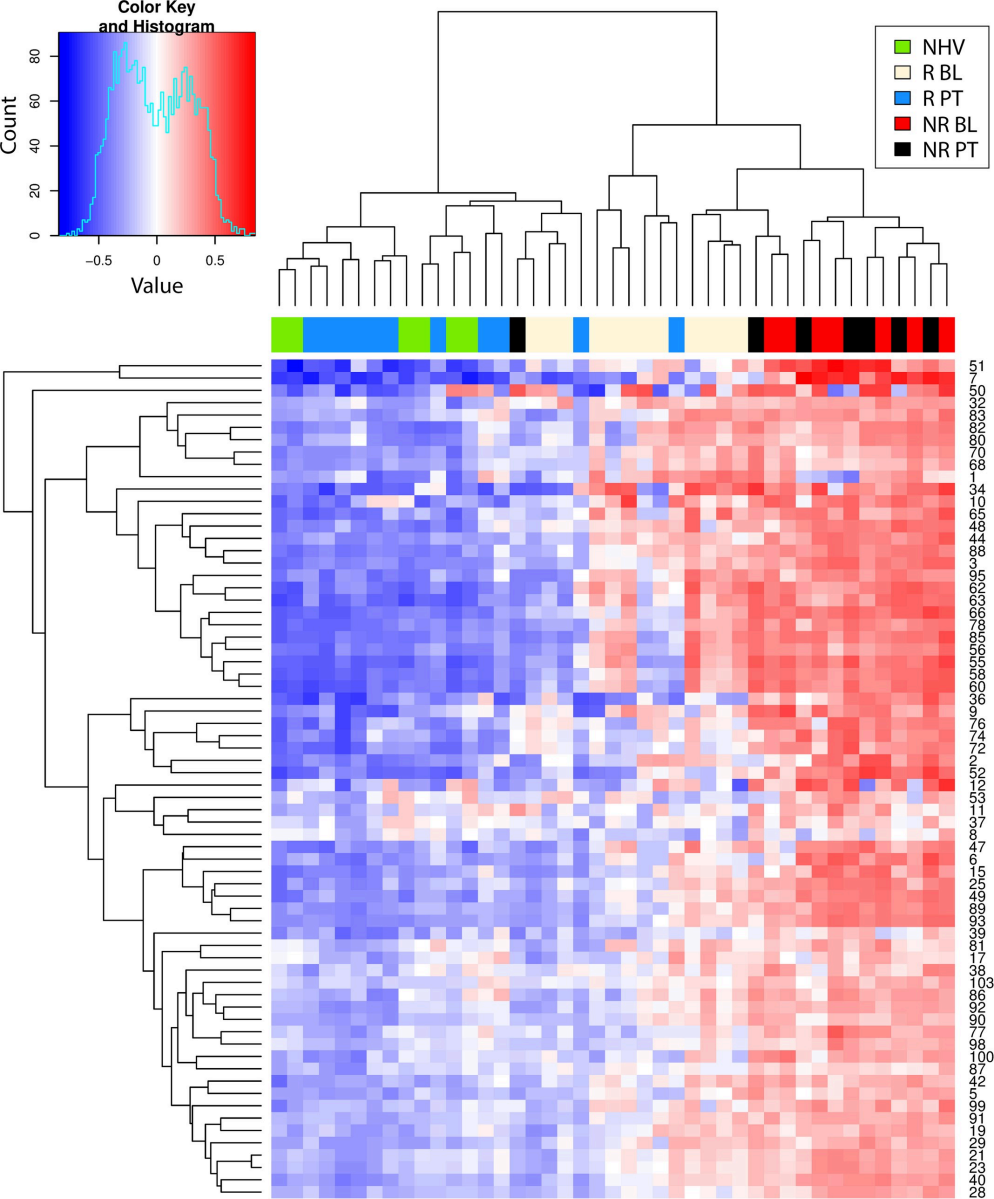
Hierarchical clustering heat map of gene set variation analysis enrichment scores of Crohn’s Disease participant samples. Shown is the heat map resulting from the hierarchical clustering of the gene set variation (GSVA) enrichment scores (ES) of Crohn’s disease (CD) participant samples (GSE16879) using all signatures significantly enriched from comparing post-treatment (PT) vs baseline (BL) in clinical responders (R) and non-responders (NR) as well as comparing R to NR at BL from Fig 2.

**Fig 4:**
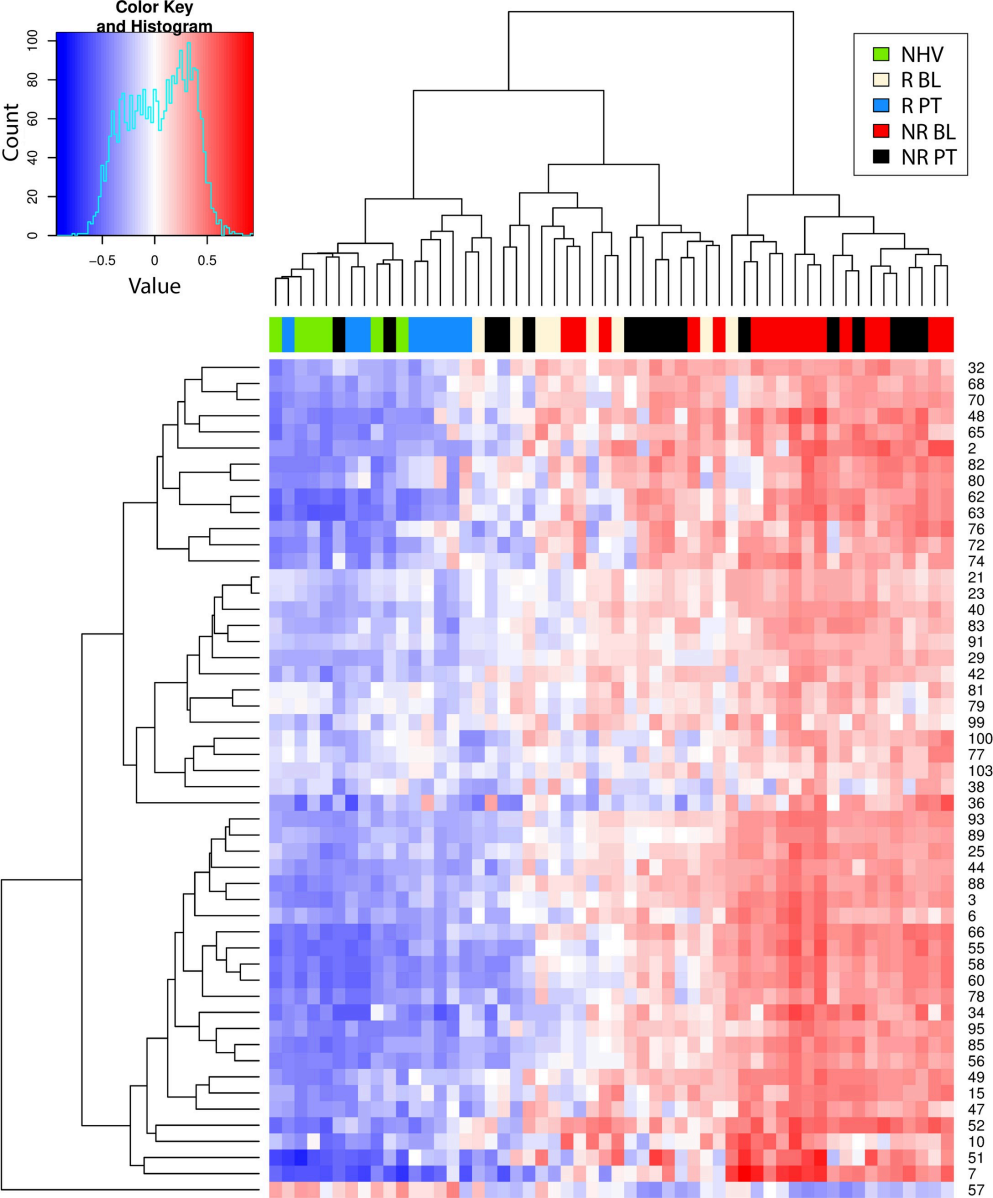
Hierarchical clustering heat map of gene set variation analysis enrichment scores of ulcerative colitis participant samples. Shown is the heat map resulting from the hierarchical clustering of the gene set variation (GSVA) enrichment scores (ES) of ulcerative colitis (UC) participant samples (GSE16879) using all signatures significantly enriched from comparing CD post-treatment (PT) vs baseline (BL) in clinical responders (R) and non-responders (NR) as well as comparing R to NR at BL from Fig 2.

Next, the molecular states (baseline and post-treatment, responder and non-responder) were mapped to a common framework to better understand how they relate. Enrichment scores for CD and UC (Figs 3 and 4, respectively) were clustered with all samples but only for the signatures that showed higher enrichment in each disease compared to healthy before treatment (Fig 2A). In CD, we obtained two main sample clusters with this approach (Fig 3). The NR population, whether at baseline or post-treatment, clustered together, consistent with our earlier observation that there were no molecular changes in this group with treatment. This cluster also included nearly all baseline R samples. Thus, we believe this cluster represents unresolved molecular disease which exists at baseline in the R population and at baseline and post-treatment in the NR population. A nearly identical cluster was observed with UC samples (Fig 4).

The second cluster was almost entirely comprised of healthy controls and nearly all R post-treatment. This suggests that while R have unresolved molecular disease at baseline, post-treatment this molecular disease has resolved such that it becomes more like healthy controls. An analysis based on clustering using whole genome expression data produced similar findings [29]. This is consistent with our earlier assertion that resolution of molecular disease, towards a healthy molecular state, is associated with clinical response. Again, a similar cluster was observed with UC samples (Fig 4).

### Differences in T, B and monocyte cells are aligned with the identified patient clusters

**Fig 5:**
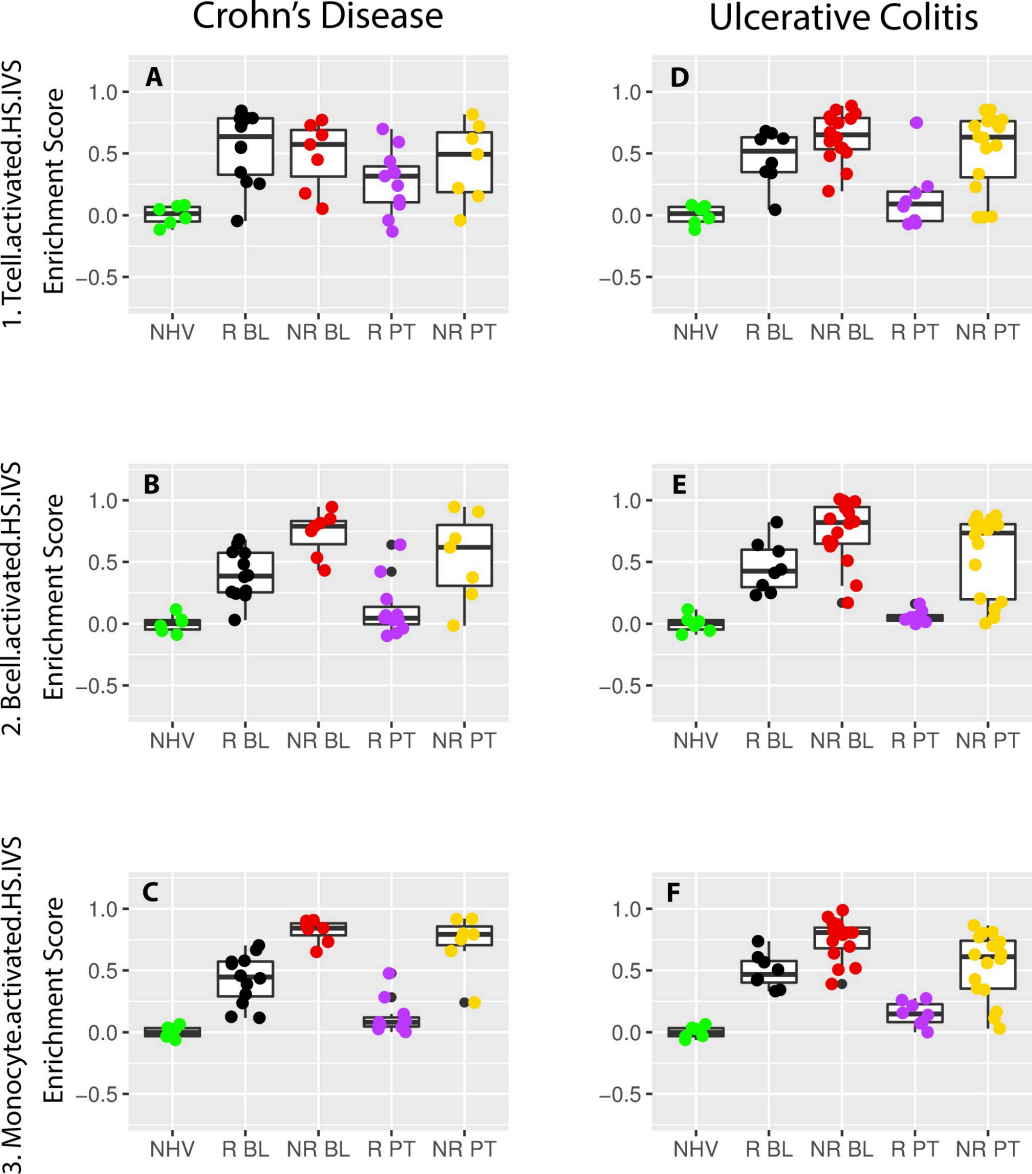
T cell, B cell and monocyte scatterplot representation of gene set variation analysis enrichment scores for the Crohn’s disease and ulcerative colitis participant samples. Shown are the scatterplots of the gene set variation (GSVA) enrichment scores (ES) of Crohn’s disease (CD) and ulcerative colitis (UC) participant samples (GSE16879) from signature #1_Tcell.activated.HS.IVS (A, D), #2_Bcell.activated.HS.IVS (B, E) and #3_Monocyte.activated.HS.IVS (C, F). Samples have been classified into normal healthy volunteer (NHV), clinical responders at baseline (R BL) or post-treatment (R PT) and clinical non-responders at baseline (NR BL) or post-treatment (NR PT). Panels A, B and C show the scores in UC while D, E and F show the scores in CD. Pair-wise T-test statistics are listed in S4 Table.

Fig 5 shows the enrichment score scatterplots for the activated T, B and monocyte signatures (#1. Tcell.activated.HS.IVS, #2. Bcell.activated.HS.IVS and #3. Monocytes.activated.HS.IVS; S1 and S2 Tables). These cell types have been described to drive inflammatory processes mediating pathology in both CD and UC [30, 26, 27]. For all three signatures, the enrichment scores at baseline were significantly higher when compared to the respective control samples in CD and UC in both R and NR. Also, an overall trend for higher enrichment in NR was observed in CD and UC with several differences being statistically significant in either or both diseases (Fig 5B–5F; S4 Table). That same trend was observed with signatures for activated dendritic cells (#5. Dendritic.activated HS.IVS), neutrophils (#6. Neutrophil.activated.HS.IVS) and macrophages (#89. Macrophages.GM-CSF.LPSc.HS.IVS) indicative of various cell types showing activation in CD and UC. However, for the activated natural killer signature (#4.NKcell.activated.HS.IVS), no differential enrichment across the patient sample groups was observed.

Post-treatment in R, no statistically significant difference was observed with healthy samples. In contrast in NR, the statistically significant difference with healthy samples was maintained. When comparing the enrichment scores from R or NR post-treatment versus baseline, all were statistically significant in R and none were in NR. A clear difference was that none of the dysregulated biology in either disease at baseline was normalized post-treatment in NR. Indeed, comparing post-treatment R versus NR, all enrichment scores comparisons were statistically significant with only one exception for activated T cells (Fig 5A).

Looking deeper into T cell biology, we next examined T helper (Th) cell and innate lymphoid cell (ILC) signatures. These cell types have been previously described as disease drivers in IBD [31, 32, 33, 34, 35, 36]. Of note, there was minimal gene composition overlap for all Th or ILC signatures (Tables 1 and S2) consistent with representing non-overlapping biology. Without separating R from NR, only the Th17 in CD and the Th1 and Th17 signatures in UC were enriched (S3 Table). When separating R from NR, none of the Th signatures were enriched in R in either CD or UC, while all of them enriched in CD and the Th1 and Th17 were enriched in UC. For the ILC signatures, the ILC2 and ILC3 signatures were enriched in all patient samples in CD while in UC the ILC1 and ILC2 were enriched. Separating samples into R and NR, none of the ILC signatures were enriched in R while all of them were in NR in both diseases. We also looked at both the intra- and inter-correlation between the T helper (Th) signatures and the innate lymphoid cell (ILC) signatures (Table 1).

**Table 1:** T helper and innate lymphoid cell signature correlations in Crohn's and ulcerative diseases.

CD			
	Th1.activated.HS.IVS.UP	Th2.activated.HS.IVS.UP	Th17.activated.HS.IVS.UP
	ID 36 (18 genes)	ID 37 (42 genes)	ID 39 (18 genes)
siLP.ILC1.MM.UP	r: 0.89, p<10–14	r: 0.70, p<10–6	r: 0.76, p<10–8
ID 100 (62 genes)	3 genes	1 gene	1 gene
siLP.ILC2.MM.UP	r: 0.60, p<10–4	r: 0.53, p<10–3	r: 0.63, p<10–5
ID 101 (123 genes)	1 gene	1 gene	3 genes
			
siLP.ILC3.MM.UP	r: 0.22, p: 0.15	r: 0.13, p: 0.42	r: 0.38, p = 0.01
ID 102 (17 genes)	1 gene	1 gene	0 genes
			
UC			
	Th1.activated.HS.IVS.UP	Th2.activated.HS.IVS.UP	Th17.activated.HS.IVS.UP
	ID 36 (18 genes)	ID 37 (42 genes)	ID 39 (18 genes)
siLP.ILC1.MM.UP	r: 0.79, p<10–15	r: 0.71, p<10–8	r: 0.64, p<10–6
ID 100 (62 genes)	3 genes	1 gene	1 gene
siLP.ILC2.MM.UP	r: 0.46, p<10–3	r: 0.57, p<10–5	r: 0.63, p<10–6
ID 101 (123 genes)	1 gene	1 gene	3 genes
			
siLP.ILC3.MM.UP	r: -0.01, p: 0.91	r: -0.14, p: 0.30	r: -0.05, p = 0.71
ID 102 (17 genes)	1 gene	1 gene	0 genes

For each pairwise comparison, the correlation (r), the p value and the number of overlapping genes are indicated. Insignificant correlations are highlighted in gray.

In CD, all signatures correlated with each other except for the ILC3 with the Th1 and Th2 signatures. Correlations R ≥ 0.7 for the ILC1 signature (#100 siLP.ILC1.MM.UP) was observed with all three T helper signatures (#36 Th1.activated.HS.IVS.UP, #37 Th2.activated.HS.IVS.UP, #39 Th17.activated.HS.IVS.UP). Similarly, the ILC2 signature (#101 siLP.ILC2.MM.UP) was also correlated with all T helper signatures although at a lower level. For the ILC3 signature (#102 siLP.ILC3.MM.UP) however the only significant correlation was observed with the Th17 signature. In UC, the ILC1 and ILC2 signatures also correlated with all T helper signatures and here also the ILC1 signature showed a stronger correlation when compared to ILC2 signature. Finally, no significant correlation was observed between the ILC3 signature and any of the T helper signatures. In conclusion, strongly correlated signatures were observed in both CD and UC between ILC1 and ILC2 signatures with all T helper signatures while the ILC3 signature only correlated in CD with the Th17 signature.

### Generation of a signature score in CD and UC

Taken together, our analysis suggests that a shared set of gene signatures differentiates CD and UC from healthy controls and that resolution of these gene signatures toward a healthy molecular state is associated with clinical response. Furthermore, for both diseases, clinical NRs are characterized by a lack of change in these gene signatures. In both diseases these observations could be explained by a tight coupling between gene signature enrichments and clinical state. These findings raise the possibility that a common set of gene signatures could theoretically be used to track the clinical state of the subject regardless of whether they have been diagnosed with CD or UC.

To test this hypothesis, we created a score by first summing the enrichment scores from the 58 upregulated signatures that were differentially expressed compared to healthy controls in both CD and UC (Fig 2A). For visual convenience, we then subtracted the mean of the healthy controls such that zero in our score would be considered a state of health. This score represents the total molecular dysregulation observed for each patient as a distance from normal with the assumption that each signature is equally important. This assumption is supported by our observation that the overlap between the genes in the signatures in the collection was very small.

**Fig 6:**
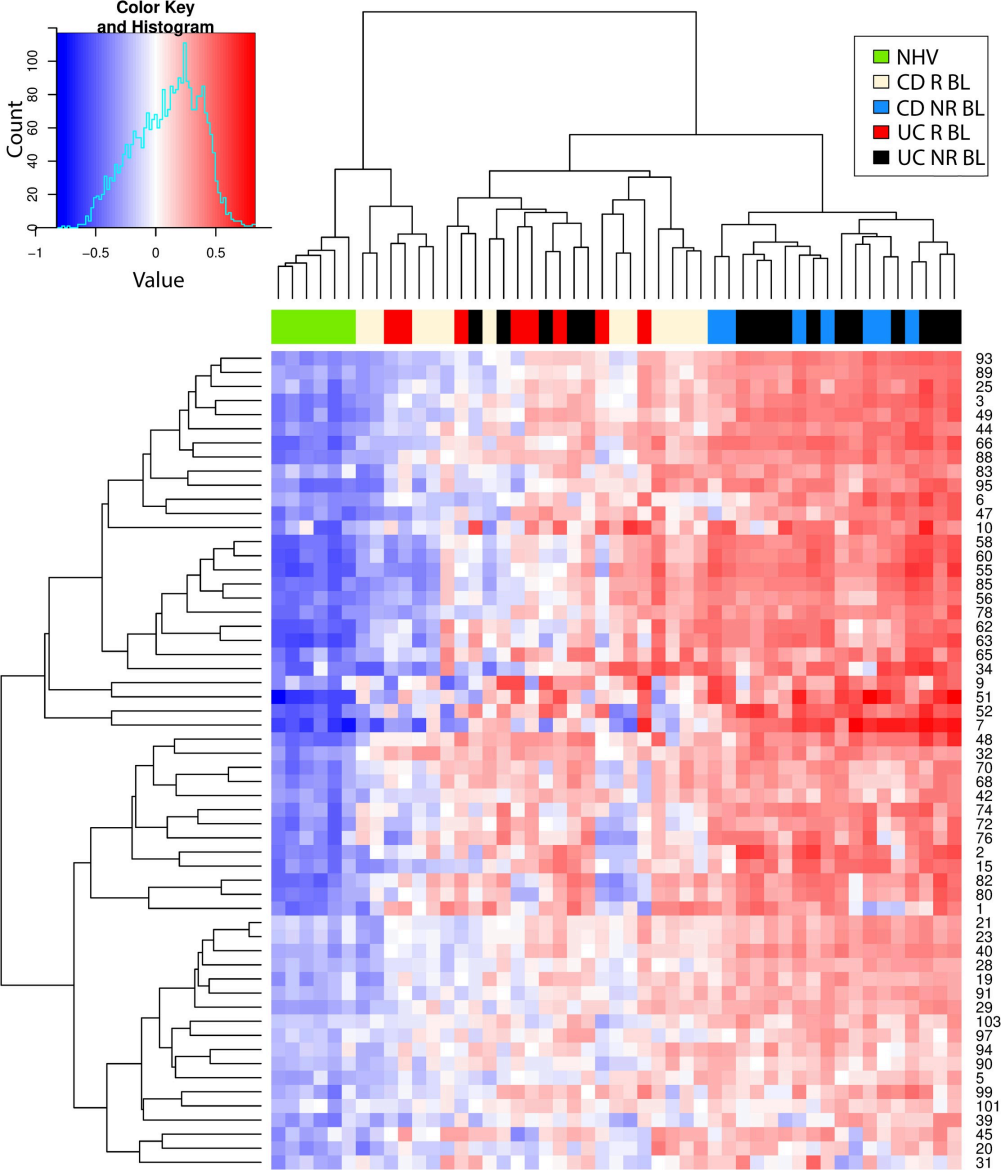
Hierarchical clustering heat map of gene set variation analysis enrichment scores of Crohn’s disease and ulcerative colitis participant baseline samples. Shown is the heat map resulting from the hierarchical clustering of the gene set variation (GSVA) enrichment scores (ES) of the Crohn’s disease (CD) and ulcerative colitis (UC) participant baseline (BL) samples (GSE16879) using all signatures significantly enriched from comparing CD or UC post-treatment (PT) vs baseline (BL) samples in clinical non-responders (NR) from Fig 2A.

**Fig 7:**
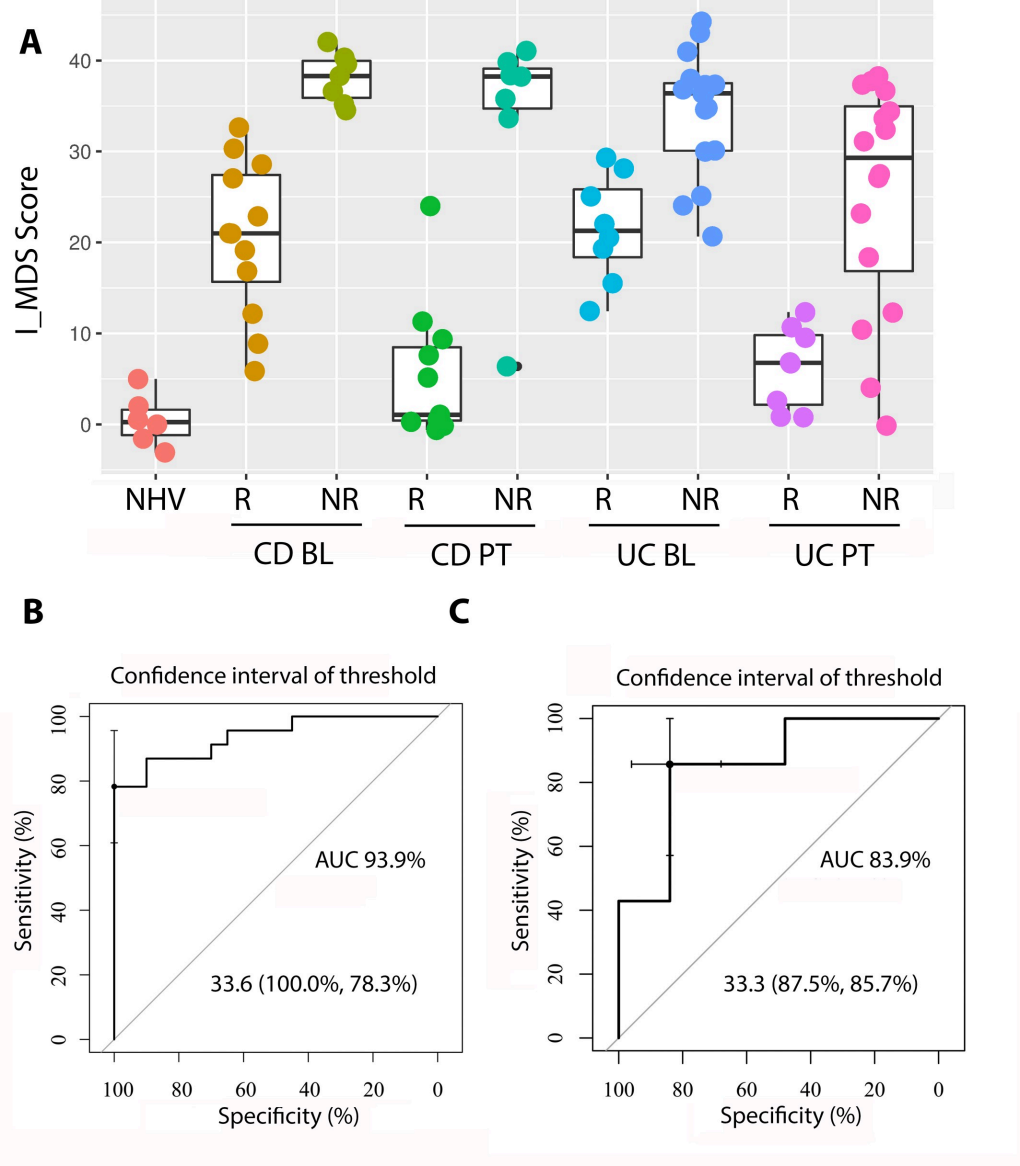
Inflammatory bowel disease molecular activity score classification of patient samples. (A) Shown are the scatterplots of the sum of gene set variation (GSVA) enrichment scores (ES) using all 58 upregulated signatures in Fig 2A common for Crohn’s disease (CD) and ulcerative colitis (UC) participant samples (GSE16879) using the following formula: I_MDS score = $\sum_{i=1}^{n}ES(CD\;and\;UC)-\frac{1}{n}*\sum_{i=1}^{n}ES(NHV)$. Samples have been classified into normal healthy volunteer (NHV), clinical responders at baseline (R BL) or post-treatment (R PT) and clinical non-responders at baseline (NR BL) or post-treatment (NR PT). Pair-wise T-test statistics are listed in S5 Table. (B) and (C) show the I_MDS score outputs identifying clinical responders and non-responders before treatment with 100% specificity and 78.8% sensitivity (B, GSE16879) and confirmation in an independent dataset [37, 38, 39] with 87.5% specificity and 85.7% sensitivity.

Fig 6 shows the clustering of the scores across the different disease groups. Clinical response is strongly associated with molecular resolution of the score post-treatment while clinical nonresponse is associated to no change in the score post-treatment. This is true for both CD and UC. Fig 7A shows the resulting scatter plot of the score across the different disease groups. As expected, the score visually discriminates disease from healthy controls. This is a trivial observation because the score only includes signatures that were differentially enriched from healthy controls. These results support the notion that molecular disease burden, as summarized by our signatures, tracks closely with clinical disease severity and could be explored as a tool for quantifying disease severity.

In addition to tracking clinical disease severity changes in time, the molecular disease severity score also appeared to differentiate R and NR populations at baseline (Fig 7A). Responders were characterized be a reduced activity score at baseline compared to non-responders, suggesting the hypothesis that response and nonresponse are related to burden of disease at baseline rather than a difference in specific pathways between the two groups. Indeed, the molecular disease severity score could identify clinical responders and non-responders before treatment with 100% specificity and 78.8% sensitivity (Fig 7B). This discriminatory ability was confirmed in an independent dataset (Fig 7C), [37, 38, 39] with 87.5% specificity and 85.7% sensitivity, suggesting a robust relationship between the score and patient outcomes.

## Discussion

We have demonstrated that by using an approach based on GSVA and signatures characteristic of inflammatory and immune processes ulcerative colitis and Crohn’s disease patient groups can be segregated by various degrees of underlying functional pathology. Signatures were commonly enriched in both conditions at baseline, supporting the notion of a disease continuum within CD and UC, however there was also heterogeneity within each disease group which was associated with the clinical response to TNF therapy. We describe an **Inflammatory bowel disease Molecular Activity Disease Score (I_MDS)** which provides a means to track the molecular state of an IBD patient, which we show is tightly coupled to the clinical state of the individual.

A typical microarray experiment when comparing groups of samples can easily result in hundreds or thousands differentially expressed genes which must then be interpreted. GSVA represents an advantage over other methods in that it computes an enrichment score from each individual sample from the observed gene expression levels, and hence is classified as single-sample method as opposed to the Over-Representation Analysis (ORA) [40] which relies on identifying differential expressed gene lists and linking them to a pathway. GSVA thus alleviates the need to select significant genes as a first step and provides a unique result for a given dataset. It also provides an estimate of the behavior of a gene set, within the entire dataset, based on the variation of other genes in the dataset, in an unsupervised manner. Thus, it does not rely on any predefined patient or sample groups such as those represented by clinical response or non-response e.g. and produces a list of enrichment scores per individual subject. It also avoids the common assumption behind the models used for ORA, such as independence between genes, a likely cause of the inflated rate of false positive findings [40]. Furthermore, the association between the phenotype and the sample-level gene set scores can be conducted with classical statistical models rendering the analysis of even very complex designs (e.g. time series, longitudinal designs, etc.) easy. Furthermore, GSVA analysis has recently been used to distinguish between subsets of psoriatic patients further highlighting the clinical utility of this bioinformatic approach [41].

Crohn's disease and ulcerative colitis are complex diseases characterized by relapsing inflammation depending upon genetic factors, deregulated immune responses, microbial dysbiosis, and environmental factors [42, 43]. While classified as separate diseases, common driving disease mechanisms have been highlighted such as the shared genetics risk factors [15, 44, 45], common gene methylation [28, 46] as well as common involvement of various biological pathways [2, 47, 39, 38]. The notion of a continuum of disorders within inflammatory bowel diseases (IBD) has also been explored [15]. Our analyses support the notion of a continuum within IBD where UC and CD samples co-cluster both at baseline and after treatment. The high level of shared disease biology illustrated by the common enrichment of 59% (61/103) of all signatures tested also supports this notion. These signatures span a wide array of biology across different cell types such as e.g. T, B, monocytes, macrophages as well as dendritic cells, including activation of a specific cell type or exposure of a cell to selected stimuli, different inflammatory pathways such as e.g. T helper cell (Th), innate lymphoid cells (ILC) or innate immunity pathways as well as signatures generated from animal models or borrowed from other diseases such as e.g. a bleomycin lung injury model or asthma. While Th1 responses have been thought to drive the pathogenesis of CD [48, 49, 50] and Th2 responses drive UC pathogenesis [48, 51, 52], our data rather suggest presence from all Th cell subsets examined especially in NR. This is in line with data published showing increased IFNγ and lower IL13 levels from patient biopsies in UC when compared to CD [53, 54] suggestive of a Th1 contribution in UC. Furthermore, dendritic cells isolated from CD and UC patients showed production of both IL17 and IL23 indicative of a Th17 involvement in both diseases as illustrated from our enrichment results of the Th17 signature in both CD and UC [55]. Increased gut mucosal IL17A transcripts as well as an increased Th17 and Th1/Th17 cell number observed in CD and UC patient samples when compared to healthy also support our findings [53, 56, 57]. Group 1 innate lymphoid cells (ILC1s) produce interferon γ and depend on Tbet, group 2 ILCs (ILC2s) produce type 2 cytokines like interleukin-5 (IL-5) and IL-13 and require GATA3, and group 3 ILCs (ILC3s) include lymphoid tissue inducer cells, produce IL-17 and/or IL-22, and are dependent on RORγt [58]. Our data show that all ILC signatures were enriched in both CD and UC in NR supporting a role of all three subsets in IBD. Indeed, an increased frequency of ILC1 and ILC3 cells in in IBD has been reported when compared to healthy [34, 59, 60, 61, 62], and this increase is associated with disease severity [63]. ILC2s producing IFNγ in addition to IL13 have also recently been described in intestinal tissues from patients with Crohn’s disease [64] in line with the enrichment of the ILC2 signature not only in all patient samples but also in NR suggesting that non-response to TNF therapy might also be linked to ILC2 activity. Highly correlated Th and ILC signature enrichments were seen in both diseases showing the tight association of these different cell types in the pathology of CD and UC. It is therefore difficult to differentiate between the roles of Th cells and their ILC counterparts in the pathophysiology of IBD. Our data advocate for equal involvement of all Th and ILC subsets where patient samples are differentiated more so by the intensity of the dysregulation of these cell subsets. These data also support the above described notion of a disease continuum in IBD rather than a UC versus CD classification and emphasize more so a difference in intensity of activation across both CD and UC across various biological mechanisms.

Neutrophils are key players of innate immunity, migrating to sites of infection to uptake and kill bacteria, releasing reactive oxygen species. However, how exactly neutrophils contribute to inflammatory bowel disease remains a controversial area. While there are studies supporting a beneficial role, others seem to point to pathological contributions [65]. It has been suggested that disturbed signal transduction activation and functionality in neutrophils, may be associated with intrinsic defects in innate immunity in CD [66]. For example, [67] have reported slower accumulation of neutrophils and delayed clearance of subcutaneously injected killed *Escherichia coli* in CD patients as opposed to controls. Such findings imply a beneficial role for neutrophils in CD. However, other experimental settings with anti-neutrophil antibodies have reported positive effect of neutrophil depletion in animal models, reducing inflammation, suggesting otherwise [68]. From our results both neutrophil signatures (#6 Neutrophil.activated.HS.IVS; #7 Neutrophil.nas.brushings.HS) were highly enriched in NR in both UC and CD indicating that activated neutrophils are part of a more severe disease activity pattern. Other innate immunity signatures e.g. the polyinosinic:polycytidylic acid (PolyIC) signatures were derived from a mouse model studying the effect of polyIC admission to lungs [69]. PolyIC is a synthetic analogue of double-stranded RNA, widely used to mimic the effects of viral infection in animal models. Here we observe that genes that were upregulated after polyIC were also correlated to NR at baseline in both diseases. These data also suggest an increased disregulation of innate immune functions in IBD linked to a more severe molecular disease phenotype as well as linked to NR to an anti-TNF therapy. In summary, the change of enrichment score of inflammatory signatures because of treatment tends to correlate with their value at baseline.

Previous gene array studies of UC [70] and CD [71] have identified non-overlapping five gene panels predictive of clinical response to infliximab. In UC, these markers separated responders from non-responders with 95% sensitivity and 85% specificity [70]. In CD patients with Crohn’s colitis were predicted with 100% sensitivity and specificity while no prediction was achieved for patients with Crohn’s ileitis [71]. The I_MSD score had comparative prediction performances with 100% specificity and 78.8% sensitivity. While neither of the gene panels were confirmed in an independent data set, the I_MSD score was confirmed in an independent data set for UC with 87.5% specificity and 85.7% sensitivity. Finally, the described gene panels are disease specific and are not described to capture the disease intensity unlike the I_MSD score.

When considering implementing the I_MSD in clinical practice, the elaboration of a co-diagnostic test would be required. For that, additional validation steps of the platform used would be required followed by a real-world usage validation in a suitable number of patients. The outcome of the test could be envisaged with two or three different recommendations for the treating physicians. Indeed, 1) the score obtained from the test is below the 32 cut off and would therefore come with an anti-TNF treatment recommendation, 2) the score would be 32 or higher and would come with a recommendation against treating with an anti-TNF or 3) a third category could be defined e.g. for scores hovering around a score of 32 where the decision would be left to the treating physician. Finally, another application of this score could be for clinical development of new therapeutics. Indeed, patients could be tested and enrolled if their score was equal or higher to 32 to select the elaboration of new therapeutics complementary to anti-TNFs.

Limitations of the current study are twofold. First, the collection of signatures assembled mainly represents inflammatory biology. While inflammation is a hall mark of IBD a more diverse set of signatures could potentially allow to identify patient groups with an increased granularity. Second, the collection of CD samples occurred in the colon only. CD is a patchy disease and collection of more than one disease location such as e.g. the ileum could also provide more granularity on the driving molecular mechanisms. Third, we cannot address whether our results are specific for a therapeutic targeting TNFα as we do not have access to data sets using a different mode of action. Finally, while a similar approach could be used for common inflammatory diseases we have not tested the I_MSD derived from our analysis in UC and CD on any other inflammatory disease treated with an anti-TNF.

### Conclusions

To conclude, a major healthcare problem is highly variable efficacy of different treatments in patients that appear to phenotypically have the same disease. This contributes to increased patient suffering, both in terms of side effects and continued disease progression due to low treatment response. An advantage of the approach presented is the ability to identify signature enrichment at the single patient level enabling patient stratification within and across diseases increasing the potential to identify patients that may respond to therapeutic agents. Indeed, we show the potential of certain signatures to be associated with treatment response through the molecular disease activity score. However, new clinical studies will need to be performed to test the clinical feasibility of using GSVA for preventative and personalized medicine approaches as seen previously in studies on glioma [72], subtypes of liver cancer [73] and for response to dual CXCR2/CCR5 therapy [69].

## Supporting information

S1 Fig Differential signatures in Crohn’s disease and ulcerative colitis across various patient group comparisons. Venn diagrams of signatures showing significantly reduced enrichment, using a general linear model analysis on GSVA ES, comparing at baseline (BL) either all (A) or clinical responder (R) (B) or clinical non-responder (NR) (C) participant samples in CD and UC. Also shown are signatures, significantly increased in R vs NR at BL (D), post-treatment (PT) vs BL in R (E) and NR (F) respectively. In A, B and C, and in D, E and F the number of signatures positively enriched are listed respectively.
(TIF)

S1 Table Gene signature collection. A table listing the collection of gene signature, along with their identifier, names, direction of gene expression, tissue and/or cell type and species from which they were derived and study identifier.
(XLSX)

S2 Table Gene signatures composition. A list of the individual genes contained in each signature.
(XLSX)

S3 Table Gene signature enrichment comparisons in the IBD dataset. Detailed listing of the signature comparison in CD, UC and NHV, for baseline and post-treatment samples, indicating statistical significance and direction of enrichment scores.
(XLSX)

S4 Table Statistical comparisons of ES scores for the activated T, B cells and monocyte signatures. Statistical significance and difference between average ES scores for the various groups in the IBD dataset GSE16789.
(XLSX)

S5 Table Statistics for Fig 7. A list of p-values for the various group comparisons indicated on Fig 7.
(XLSX)
