# Supplementary figures and images for: I_MDS: an inflammatory bowel disease molecular activity score to classify patients with differing disease-driving pathways and therapeutic response to anti-TNF treatment
Source: PLoS Comput Biol. 2019 Apr 30;15(4):e1006951. doi: 10.1371/journal.pcbi.1006951 (PMC6510457; doi:10.1371/journal.pcbi.1006951)

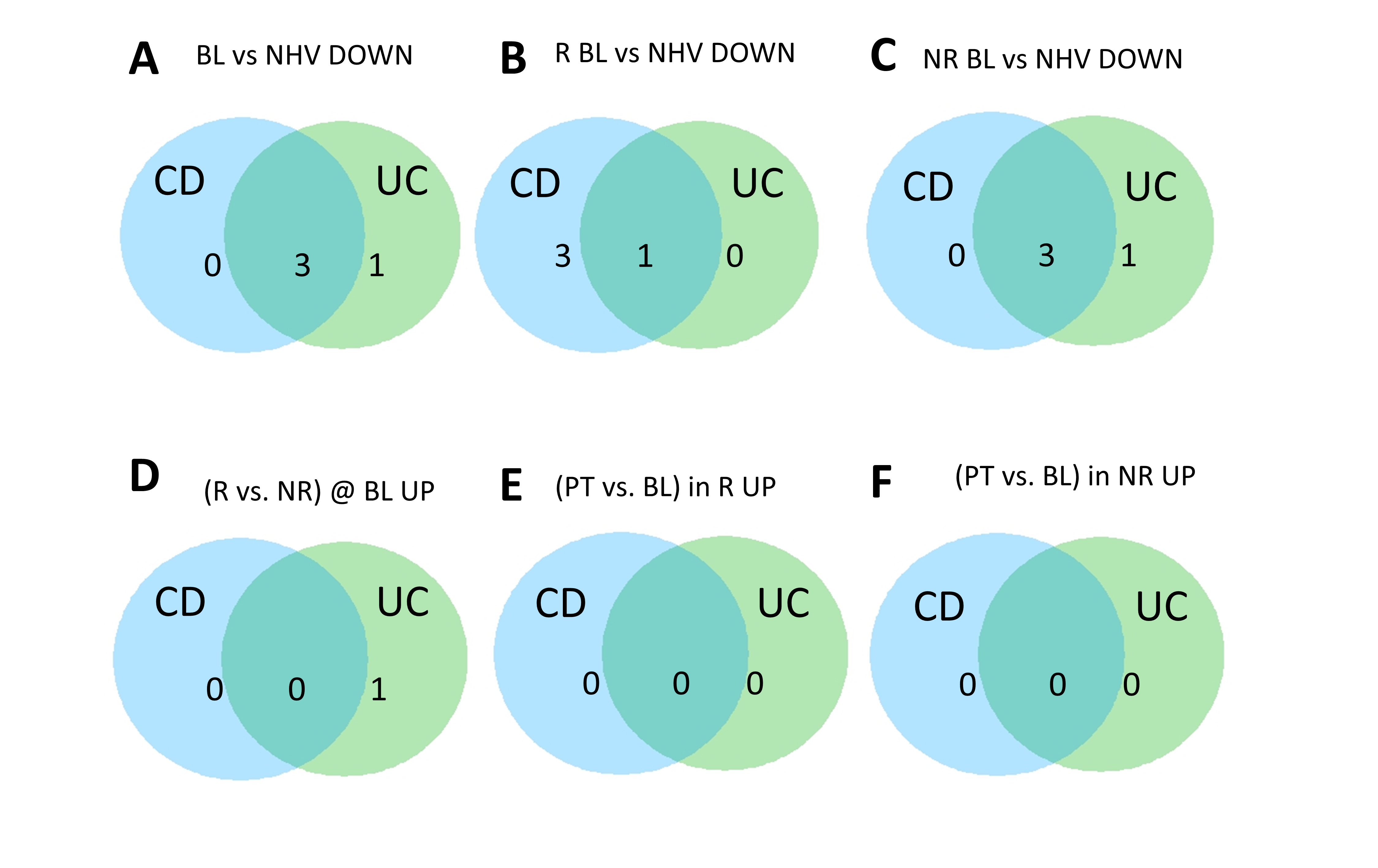

Supplement: S1 Fig — Venn diagrams of signatures showing significantly reduced enrichment, using a general linear model analysis on GSVA ES, comparing at baseline (BL) either all (A) or clinical responder (R) (B) or clinical non-responder (NR) (C) participant samples in CD and UC. Also shown are signatures, significantly increased in R vs NR at BL (D), post-treatment (PT) vs BL in R (E) and NR (F) respectively. In A, B and C, and in D, E and F the number of signatures positively enriched are listed respectively. (TIF) [file pcbi.1006951.s001.tif]
